# Editorial: Cross-Cultural and Relational Views on Nature

**DOI:** 10.3389/fpsyg.2022.937051

**Published:** 2022-07-27

**Authors:** Susana Alves, Gowri Betrabet Gulwadi, Paula Villagra

**Affiliations:** ^1^Department of Psychology of Developmental and Socialization Processes, Sapienza University of Rome, Rome, Italy; ^2^School of Applied Human Sciences, University of Northern Iowa, Cedar Falls, IA, United States; ^3^Instituto de Ciencias Ambientales y Evolutivas, Facultad de Ciencias, Austral University of Chile, Valdivia, Chile

**Keywords:** culture, natural environments, relational view of nature, biophilia, human-nature connectedness, other-than-human nature

Since the launch of this Frontiers Research Topic, two world events–the COVID pandemic and the Ukrainian war have affected our lives. These examples of disconnection—being locked indoors (COVID-19 situation) or forced into displacement due to war—have intensified our need for relatedness and contact with nature. This collection of four articles brings to the fore a need for an integrative view of nature, and an important cross-cultural factor: humans' need for relatedness and connectedness to nature.

In the first article, Alves et al. propose an integrative conceptual framework to examine human-nature connectedness in campus settings. From a relational point of view, biophilia—humans' innate need to affiliate with nature—creates a structuring device to understand how university students connect to nature, others, and themselves through direct/indirect and place-based experiences in nature. Such a long-term perspective enables us to formulate practical strategies for enhancing connectedness in campus settings by integrating Kellert's biophilic design principles with Alexander's pattern language.

Blanco-Wells writes that the current environmental crisis must compel us to critically address the ontological separation between humans and nature. In this second article, a relational view of nature encompasses the entanglement between human and non-human agents–that is, the need to consider other-than-human natures. Blanco-Wells presents *ecologies of repair* as a heuristic device to understand and transform human and non-human life forms. His experimentation with socio-geo-ecologies is exemplified with research in Southern Chile.

Moving to the local level,  Paniotova-Maczka et al. discuss place attachment as a relational variable to examine how Polish residents in rural and urban municipalities view tree management. Place attachment in the form of “public good sentiment” and the perception of ecosystem services provided by trees related to private interests significantly impacted residents' views on tree management. Place attachment may work as a “relational tool” to manage trees in a way that avoids social conflicts and inter-cultural disparities.

Finally, Wang questions the utility of current models to explain the “self” in different cultural backgrounds. He contrasts two different notions of “self” in Western and Eastern cultures that can influence how humans relate to nature. The subject-object dichotomy in the Western notion of self is contrasted with the oneness model of self in Chinese Buddhism. Wang highlights the “one mind-two aspects self-model” as a valuable approach to understand different cultural contexts, where society, humans, and nature are constantly changing.

Overall, the four articles help us unravel different facets of how humans relate to nature. An innate and evolutionary tendency for affinity with nature is contrasted with ontological views regarding human nature and other-than-human nature. Together they create intriguing glimpses into ways that we might frame our inquiry in the future, especially if we are to better understand what may be distinctive and what may be pervasive across different cultures. This seems to take on greater relevance within the most recent global pandemic and war context, where the relationship between humans, societies, and nature has been forced to change. Also, this set of articles helps us question our cultural notions of self. Doing so might reveal why we preserve or destroy nature.

## Author Contributions

SA contributed to the conception and first draft of the editorial. All authors contributed to editorial revision, read, and approved the submitted version.

## Conflict of Interest

The authors declare that the research was conducted in the absence of any commercial or financial relationships that could be construed as a potential conflict of interest.

## Publisher's Note

All claims expressed in this article are solely those of the authors and do not necessarily represent those of their affiliated organizations, or those of the publisher, the editors and the reviewers. Any product that may be evaluated in this article, or claim that may be made by its manufacturer, is not guaranteed or endorsed by the publisher.

